# Memory for stimulus sequences: a divide between humans and other animals?

**DOI:** 10.1098/rsos.161011

**Published:** 2017-06-21

**Authors:** Stefano Ghirlanda, Johan Lind, Magnus Enquist

**Affiliations:** 1 Department of Psychology, Brooklyn College, Brooklyn, NY, USA; 2 Departments of Psychology and Biology, Graduate Center of the City University of New York, New York, NY, USA; 3 Centre for the Study of Cultural Evolution, Stockholm University, Stockholm, Sweden; 4 Department of Zoology, Stockholm University, Stockholm, Sweden

**Keywords:** stimulus sequences, working memory, animal cognition, human uniqueness

## Abstract

Humans stand out among animals for their unique capacities in domains such as language, culture and imitation, yet it has been difficult to identify cognitive elements that are specifically human. Most research has focused on how information is processed after it is acquired, e.g. in problem solving or ‘insight’ tasks, but we may also look for species differences in the initial acquisition and coding of information. Here, we show that non-human species have only a limited capacity to discriminate ordered sequences of stimuli. Collating data from 108 experiments on stimulus sequence discrimination (1540 data points from 14 bird and mammal species), we demonstrate pervasive and systematic errors, such as confusing a red–green sequence of lights with green–red and green–green sequences. These errors can persist after thousands of learning trials in tasks that humans learn to near perfection within tens of trials. To elucidate the causes of such poor performance, we formulate and test a mathematical model of non-human sequence discrimination, assuming that animals represent sequences as unstructured collections of memory traces. This representation carries only approximate information about stimulus duration, recency, order and frequency, yet our model predicts non-human performance with a 5.9% mean absolute error across 68 datasets. Because human-level cognition requires more accurate encoding of sequential information than afforded by memory traces, we conclude that improved coding of sequential information is a key cognitive element that may set humans apart from other animals.

## 1. Introduction

Human achievements such as complex societies, art and science are ultimately grounded in cognitive abilities that other species lack. Paradoxically, however, researchers have struggled to identify cognitive elements that are uniquely human. Overall, the search for uniquely human cognition has focused on how different species process information [1], but less on how information is initially encoded or represented [2–5]. Here, we focus on the encoding of sequential information, showing that available evidence supports the hypothesis that non-human animals do not faithfully encode the succession of stimuli they experience.

Even a cursory survey of cognitive domains in which humans excel (table 1) reveals that correct representation and processing of sequential information is crucial to human cognition [3,6]. For example, *whale killer*and *killer whale* have different meaning in English, and indeed all languages use word order to convey information [7]. Language, as well as the imitation of action sequences, also requires correct encoding and recall of sequential information. Both are daunting tasks for non-human animals [8–10]. Remarkably, human sequence processing is not restricted to a specific context, set of stimuli or sensory modality. For instance, languages can be spoken, written, signed, embossed as Braille and even whistled [11]. Although here we are primarily concerned with sequences of stimuli, it is noteworthy that sequential structure is also critical to our mental life, e.g. in recollecting histories of events, in problem solving and in planning for the future.

**Table 1.** Role of sequential information in human abilities. The examples are not meant to be mutually exclusive. For example, episodic memory may be involved in planning, theory of mind and so on. The goal of these examples is to illustrate the pervasive importance of accurate sequential information.

ability	role of sequential information	example
episodic memory	enables ordering of events	I arrived first ≠ You arrived first
causal learning	enables attribution of cause and effect	Noise causes prey to escape ≠ Prey escaping causes noise
planning	plans are ordered sequences of actions	Turn door handle, then push ≠ Push door, then turn handle
imitation	order of actions must be perceived and remembered	She peeled the banana, then ate it ≠ She ate the banana, then peeled it
language	contributes to meaning	Killer whale ≠ whale killer
social intelligence (theory of mind)	enables attribution of beliefs and knowledge to individuals	You spoke to her before me ≠ You spoke to me before her
cooperation	order of actions must be agreed upon and remembered	I count to three, then we lift ≠ We lift, then I count to three
music	order determines aesthetic qualities, e.g. melody	CDEC ≠ CECD
mathematics	order of operations influences results	3−5≠5−3

At first sight, the claim that animals encode sequential information poorly seems to clash with many observations. For example, hearing a bell *before* receiving food will lead a dog to salivate to the bell, but hearing the bell *after* receiving the food will not. However, this discrimination (and similar ones in associative learning) only requires remembering what happens before a biologically salient event (food), rather than the representation of arbitrary sequential information. Other behaviours, such as echolocation in bats [12] and song learning in birds [13], involve genuinely complex sequence processing, but are also likely to rely on task-specific adaptations. Here, we are concerned with studies in which animals have been trained to discriminate sequences of stimuli for which they have no specific biological adaptation. For example, animals may be trained to respond to a sequence AB (stimulus A followed by stimulus B) while ignoring B alone, or to respond to AB while ignoring AA, BA and BB.

To avoid confusing difficulties in sequence processing with perceptual or procedural difficulties, we chose to include only studies that meet two requirements. First, the stimuli used to compose sequences should be readily identifiable by the animals. Selected studies fulfil this requirement in either of two ways: either the stimuli come from the animals’ natural repertoire (e.g. own species vocalizations), or the stimuli are known to be readily identifiable from previous research. For example, rats and pigeons do not have dedicated adaptations for processing such stimuli as monochromatic lights, white noise or pure tones, yet research shows that they identify these stimuli and associate them with experimental outcomes.

We also required studies to employ experimental paradigms known to be readily mastered when single stimuli are used, rather than sequences of stimuli. For example, pigeons readily learn to obtain food by pecking or not pecking in response to single stimuli, hence we have included studies training pigeons to peck or not peck in response to *sequences* of stimuli. In summary, by imposing these requirements on stimuli and experimental procedures, we are reasonably sure that, whenever an animal fails to solve a discrimination, the reason should be sought in the sequential nature of the task rather than in a poor choice of stimuli or task.

## 2. Stimulus sequence discrimination across species

**Figure 1.**
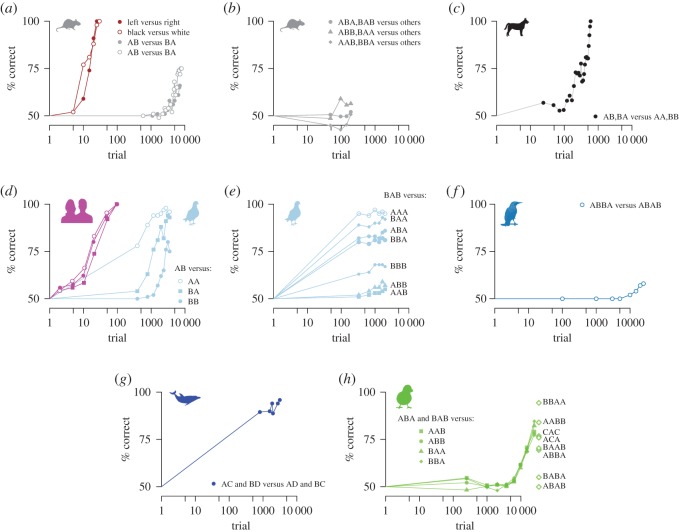
Sequence discrimination in humans and non-human animals. Labels of the form ‘*X* versus *Y* ’ identify as *X* the rewarded sequences and as *Y* the unrewarded ones. The vertical axis (% correct) gives the fraction of responses emitted to rewarded sequences. (*a*) Acquisition of a discrimination between two acoustic sequences in rats (AB versus BA, each repeated five times, two experiments [14,15]); also shown is the much faster acquisition of two A versus B discriminations (go left versus right, choose black versus white [16]). (*b*) Acquisition, in rats, of three discriminations formed using sequences in the set AAB, ABA, ABB, BAA, BAB, BBA [17]. (*c*) Discrimination in dogs between two rewarded acoustic sequences, AB and BA, and two unrewarded sequences AA and BB [18]. (*d*) Comparison of performance on the same task in pigeons (light blue [19]) and humans (magenta, electronic supplementary material, S3). (*e*) Discrimination in pigeons between three-stimulus visual sequences [20]. (*f*) Acoustic sequence discrimination in starlings: 16 AABB versus 16 ABBA sequences [21]. (*g*) Discriminations between acoustic sequences in a dolphin [22]. (*h*) Discrimination between song syllable sequences in zebra finches [23]. Open diamonds give responding to untrained sequences tested after training. For these sequences, ‘50% correct’ means responding equal to that of trained sequences; ‘100% correct’ means no responding.

**Figure 2.**
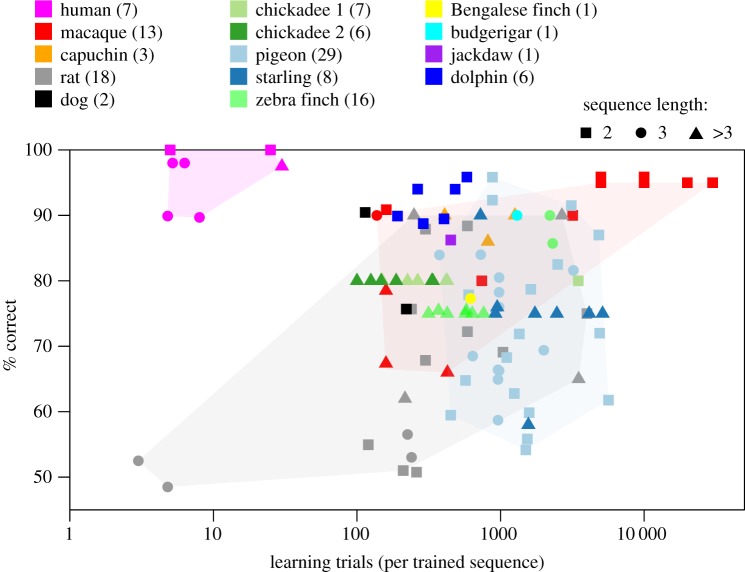
Eventual performance versus amount of training in all reviewed studies (electronic supplementary material, S1). Colour indicates species; point shape indicates length of sequences to which a response was trained. Shaded areas show the range of values spanned by data for the most studied species. Numbers in parenthesis refer to the number of experiments for each species. Chickadee 1 indicates black-capped chickadee; chickadee 2 indicates mountain chickadee. Total number of studied individuals varies from more than 100 pigeons and rats to a single dolphin.

Figure 1 shows representative learning curves from sequence discrimination studies. A first, evident conclusion is that sequence discriminations are much harder than discriminations between single stimuli. Rats, for example, can discriminate perfectly between two stimuli in 10–50 trials (provided the stimuli are not too similar), but can require thousands of trials to discriminate between two sequences (figure 1*a*). Figure 2 shows a summary of all reviewed studies in terms of the amount of training required to reach a given level of performance. A linear mixed model of discrimination performance, with study as random effect to control for task differences, reveals a significant effect of trials (*χ*^2^(1)=34.2, *p*<10^−8^) but no effect of species (*χ*^2^(5)=7.23, *p*=0.20) and no interaction between species and trials (*χ*^2^(3)=0.95, *p*=0.81), suggesting no large species differences in the speed of sequence discrimination learning (see electronic supplementary material, S1 for data sources).

The discriminations in the reviewed studies are trivial for humans and are seldom studied. Human studies, in fact, typically employ sequences of 5–10 elements, often presented just one or a few times [24,25]. However, some studies do compare humans and other species on the same task. In two studies comparing Bengalese finches [26] and zebra finches [27] to humans, the birds required 300–800 trials/sequence to achieve 80–90% accuracy on discriminations that took humans 6–8 trials/sequence to learn to 90–98% accuracy, even when the sequences were composed of Bengalese or zebra finch song syllables unfamiliar to humans. In another study [28], humans took 30 trials/sequence to reach 90% correct on a discrimination that took macaques 400 trials/sequence to learn to 70% accuracy. Lastly, we replicated a study [19] that trained pigeons to discriminate between a sequence AB (reinforced) and sequences AA, BB and BA (figure 1*d*; electronic supplementary material, S3). In our replication, humans reached over 95% accuracy within 25 trials/sequence, while in the original study pigeons took 1000 trials/sequence to learn to 85% accuracy.

Comparing human and non-human data may harbour several potential confounds. First, the stimuli used may be more easily discriminated by humans than by the animals. This seems unlikely in the reviewed studies, which used bird song syllables [26,27], distinctly coloured lights [19] (pigeons’ colour vision is superior to humans’), or simple tones [28] differing by ∼50 Hz in a frequency range in which the subject species (rhesus macaques) can discriminates differences of ∼5 Hz [29]. Second, humans may benefit from verbal instruction. Animals, however, generally receive extensive pre-training with the apparatus, which itself consists of hundreds or thousands of training trials (which have not been included in our analysis of learning times). At the start of a discrimination task, therefore, animals are very familiar with the experimental apparatus. Task instructions, moreover, typically deal with the mechanics of the task, i.e. that it is possible to produce a response and get feedback about its correctness. They do not reveal what the correct response is. In the reviewed studies, the only exception is a comparison of humans and macaques [28], in which humans were instructed to press a button upon hearing tone sequences falling in pitch. The monkeys, however, were already familiar with a similar task in which they had to discriminate tone sequences differing in pitch pattern [30].

Figure 2 shows large variation in performance for a given number of learning trials, even within species. We have investigated the sources of this variation using linear mixed models to relate discrimination performance to structural and temporal aspects of sequences (table 2). We found that discrimination is more difficult between sequences that end with the same stimulus (figure 1*d*,*e*), and even more difficult when the last two stimuli are the same (figure 1*e*). Additionally, we confirmed observations in the literature that lengthening blank intervals between stimuli or at the end of sequences has detrimental effects on performance. Conversely, lengthening the duration of stimuli and inter-trial intervals improves performance.

**Table 2.** Effect of sequence structure and temporal parameters on discrimination performance. Effect is measured as estimated change in the percentage of correct responses. Significance is assessed by analysis of deviance of linear mixed models (*χ*^2^ statistic with one degree of freedom). The first two lines refer to a linear mixed model with % correct responses as the dependent variable, two-fixed effects variables (identity of the last stimulus in the to-be-discriminated sequences, and identity of the last two stimuli) and experiment as a random effect to control for differences in experimental tasks. The analysis is restricted to studies employing sequences of two or three stimuli. The following lines estimate the effect of temporal parameters. These effects refer to a 1 s increase in the independent variable. For example, an increase in stimulus duration of 1 s is estimated as improving correct responses by 2%. All models are linear mixed models restricted to studies in which the variable of interest was varied, with experiment as a random effect.

	effect	*χ*^2^(1)	*p*<
*sequence content*:			
same last stimulus	−8%	43.4	10^−9^
same last two stimuli	−18%	83.7	10^−9^
*duration of*:			
stimuli	2%	26.0	10^−6^
interval between trials	0.2%	7.7	0.01
gap between stimuli	−1%	162.4	10^−9^
gap between end of sequence and response opportunity	−2%	14.9	0.001

Our results do not negate that animals are capable of sophisticated processing of sequences for which specific adaptations have evolved [12,13]. Rather, they show that non-human animals have a limited ability, compared with humans, to discriminate arbitrary stimulus sequences. This conclusion is strengthened by the following observations, some of which have been anticipated above. The studies we reviewed trained simple instrumental discriminations that are readily mastered when they involve single stimuli rather than sequences (figure 1*a*). The stimuli used are themselves readily discriminable (high- and low-pitch tones, dark versus light, etc.). Some studies, indeed, used naturalistic stimuli which animals should be well equipped to process, such as conspecific vocalizations [23,26,27]. Crucially, animals may solve readily some discriminations while having great difficulty with others, even within the same experiment. For example, in the experiment in figure 1*e* pigeons discriminated BAB from AAA to 95% accuracy in about 300 trials, while showing almost no improvement in discriminating BAB from AAB for about 2000 trials. In these cases, the difficulty clearly lies in the temporal structure of sequences, rather than in a poor choice of task or stimuli.

## 3. Model of non-human sequence memory

**Figure 3.**
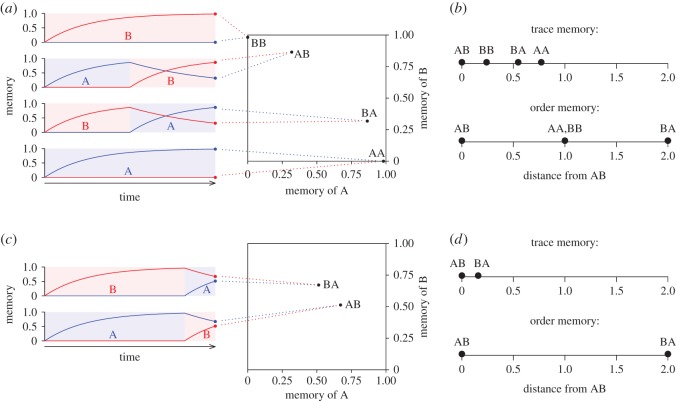
Representation of stimulus sequences through memory traces. (*a*) Representation of sequences of two stimuli of equal duration (AA and BB indicate the repetition of the same stimulus). Graphs on the left show the dynamics of memory traces. Blue lines represent the memory of A, red lines of B. Shaded areas indicate when each stimulus is present. The right panel shows how the traces map into a two-dimensional memory space. We hypothesize that the difficulty of a discrimination is inversely proportional to distance in this space. (*b*) Comparison of a trace memory with a toy model of memory with a strict notion of order (see text for details). (*c*) Representation of AB and BA sequences in which the first stimulus is much longer than the second. The memory traces are closer than in (*a*), assumed to correspond to a more difficult discrimination. (*d*) Comparison of a trace memory and an order memory relative to the discrimination of the sequences in (*c*).

Several authors have suggested that non-human sequence discrimination may rely on simple memory traces, that is, on ‘impressions’ of stimuli that fade progressively after stimulus removal [31,32]. For example, AB and BA could be distinguished based on the fact that the first sequence results in a stronger trace of B and a weaker trace of A. Here, we formulate a mathematical model based on this intuition, and we evaluate it quantitatively. We define the trace of stimulus *S* at time *t* as a single number, *m*_*S*_(*t*). When *S* is present, *m*_*S*_(*t*) increases towards a maximum value of 1, while when *S* is absent *m*_*S*_(*t*) decreases towards a value of 0. We assume that *m*_*S*_(*t*) follows these differential equations:

3.1 $$m_{S}^{\prime }(t)=\left\{ \begin{array}{ll} r_{\mathrm{up}}[1-m_{S}(t)] & \text{if }\;S\;\text{ is present},\\ -r_{\mathrm{down}}m_{S}(t) & \text{if }\;S\;\text{ is absent}, \end{array}\right.$$

where *m*′_*S*_(*t*) is the time derivative of *m*_*S*_(*t*), and *r*_*up*_ and *r*_*down*_ are the rates of memory increase and decrease, respectively. Equation (3.1) can be readily solved (electronic supplementary material, S2), enabling us to calculate the memory trace of any sequence of stimuli. Example memory traces are shown in figure 3*a* for four sequences of two stimuli of equal duration, i.e. AA, AB, BA and BB. The figure also shows how the sequences are mapped onto four representative points in a two-dimensional memory space with the trace strength of A and B as coordinates. That is, the representation at time *t* of a sequence of *A* and *B* is the pair (*m*_*A*_(*t*),*m*_*B*_(*t*)) of the memory traces of A and B at *t*. The points in figure 3*a* correspond to the time at which the sequences end.

The main idea behind our model is that the discriminability of two stimulus sequences depends on how far the sequence representations are at the time the decision is made, i.e. at the time animals are required to choose between responding or not responding. For example, the relative distances of AA, BB and BA from AB in figure 3*b* suggest that discriminating AB from BB should be difficult, because these two sequences are represented close to each other. AB versus BA should be an easier discrimination, and AB versus AA should be even easier. Empirical data show precisely this ordering (figure 1*c*) [19,33]. Note that not all memory representations would lead to the same conclusion. For example, figure 3*b* also shows distances between memories in a toy model of a memory with a strict representation of order. We refer to this model as the ‘order memory’. This memory has four bits. Bits one and two encode the presence (1) or absence (0) of A and B, respectively, in the first position in the sequence; bits three and four encode the presence or absence of A and B in the second position. Thus AB is represented as {1,0,0,1}, while AA as {1,0,1,0}. As shown in figure 3*b*, in the order memory the sequences AA, BB, AB and BA are represented further apart, hence, according to our hypothesis, discriminations should be easier than in the trace memory. Figure 3*c*,*d* shows how the two memories are affected if the first stimulus is much longer than the second; the difficulty of discriminations increases in the trace memory, but not in the order memory. (Although the order memory is intended primarily as an illustration of how memory representation may affect the difficulty of discriminations, we also note that it is a better match than the trace model to the human data in figure 1*d*, which show no great difference in learning speed across discriminations.)

Equation (3.1) is the core of our sequence discrimination model. To compare the model to data, however, we also need assumptions on how memory representations are used in decision-making, which we keep as simple as possible. Consider a discrimination between one rewarded sequence, *p*, and one non-rewarded sequence *n*, and let *d*(*p*,*n*) be the Euclidean distance between their representations at the time the decision is made. Intuitively, the discrimination should be easier the larger *d*(*p*,*n*) is. If *R*(*p*) and *R*(*n*) indicate the strength of responding to *p* and *n* after a period of training, we can formalize this intuition by writing

3.2 $$\frac{R(p)}{R(p)+R(n)}=\frac{1}{2}+cd(p,n),$$

where the left-hand side is the proportion of correct responses (the quantity graphed in figure 1 as a percentage) and *c* is a positive parameter. According to equation (3.2), discrimination performance is at chance level ($\frac{1}{2},$ or 50% correct) for sequences that are represented as identical (*d*(*n*,*p*)=0), and increases with distance between representations. (We assume that a value of equation (3.2) equal to or greater than one corresponds to perfect discrimination.) When there are multiple rewarded and non-rewarded sequences, equation (3.2) is not sufficient, but can be generalized by writing the response *R*(*x*) to a generic sequence *x* as

3.3 R(x)=g+h[⟨d(x,n)⟩−⟨d(x,p)⟩],

where *g* and *h* are constants, and 〈*d*(*x*,*n*)〉 and 〈*d*(*x*,*p*)〉 are the average distances of *x* from all non-rewarded and rewarded sequences. Equation (3.3) reflects the simple assumption that responding is an increasing function of proximity to rewarded sequences, and of distance from non-rewarded sequences. If only one rewarded sequence and one unrewarded sequence are considered, equation (3.3) yields, taking into account that *d*(*p*,*p*)=*d*(*n*,*n*)=0 and *d*(*p*,*n*)=*d*(*n*,*p*),

$$\begin{array}{rl} R(p) & =g+hd(n,p)\\ R(n) & =g-hd(n,p) \end{array}$$

from which equation (3.2) is recovered with *c*=*h*/2*g*.

**Figure 4.**
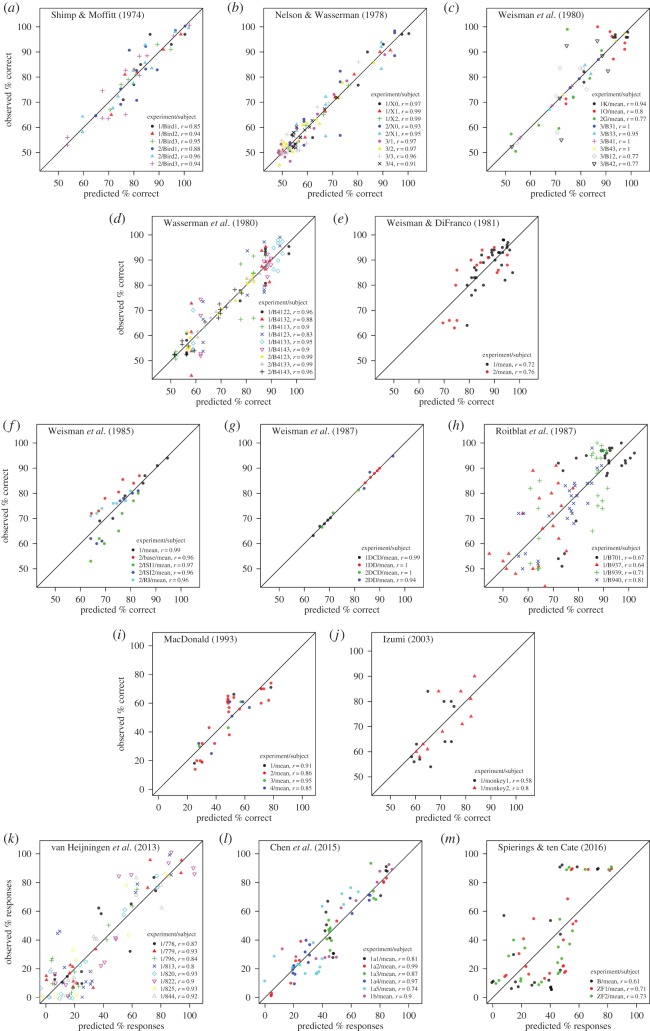
Prediction of sequence discrimination performance with the memory trace model (figure 3). We fitted individual data when available, otherwise mean group data (*l*) displays mean group data for legibility, but individual fits are similar (see electronic supplementary material, S1 for study information). In (*i*), chance performance was 16% correct rather than 50%. Study species: (*a*–*i*), pigeons; (*j*), rhesus macaques; (*k*–*m*), zebra finches; (*m*) budgerigars. Panels (*k*–*m*) include test stimuli to which a response was not explicitly trained (cf. figure 1*h*).

**Figure 5.**
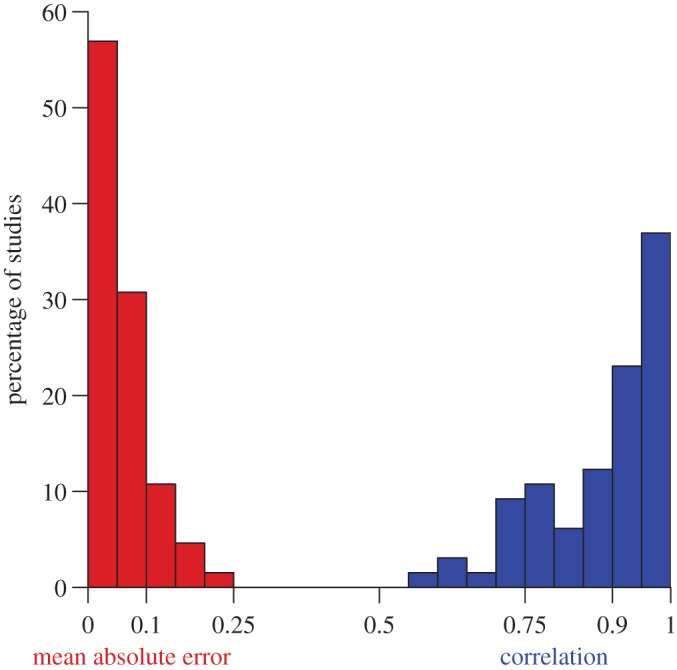
Distribution of mean absolute errors (MAEs, red) and Pearson’s correlations (blue) between predicted and observed performance across the 68 fitted datasets in figure 4. MAEs refer to the average, per dataset, of |*R*(*x*_*i*_)−*R*_*i*_|, where *x*_*i*_s are tested stimulus sequences, *R*_*i*_ is the observed response to *x*_*i*_ and *R*(*x*_*i*_) is the response predicted by a fitted memory trace model (see electronic supplementary material, S2). Responding is normalized in the interval [0,1], so that an MEA of 0.1 signifies a 10% average error.

We evaluate the model given in equations (3.1) and (3.3) by fitting the memory parameters *r*_up_ and *r*_down_ to maximize the correlation between observed discrimination performance and model predictions (*g* and *h* are also fit, but this merely serves to bring *R*(*x*), which is based on arbitrary units of distance, within the range [0,1], as is necessary to model discrimination performance (see electronic supplementary material, S2). We use the same *r*_up_ and *r*_down_ for all stimuli in an experiment. That is, although it is conceivable that the memories of different stimuli build up and decay at different rates, we adopt here the assumption that these rates are the same for all stimuli used in an experiment. Across experiments, we fit different values of *r*_up_ and *r*_down_ to account for differences in experimental details that are known to influence animal working memory [34]. Additionally, we use a third parameter, *r*_blank_≤*r*_down_, during inter-stimulus and inter-trial intervals, based on the assumption that memory decay is slower when external stimulation is minimized. We found 68 datasets providing information sufficient to evaluate the relative difficulty of a set of discriminations (figure 4). These data are representative of all the effects reported in table 2, and include variation in stimulus duration (range: 0.1–12 s), in inter-trial intervals (5–60 s), in gaps between stimuli (0–50 s), and in gaps between sequence end and the opportunity to respond (0–50 s). The fit between the trace model and the data is impressive, with a mean Pearson’s correlation between predicted and observed responding of 0.88 and a mean absolute error per dataset of 5.9% (figure 5).

Crucially, the trace model successfully reproduces effects of sequence structure that would not be expected if sequential information were faithfully encoded. For example, the discrimination between AB and BA is predicted to be more difficult when the first stimulus is much longer than the second than when the stimuli have the same duration (figure 3). Indeed, MacDonald [32] found ∼50% accuracy after ∼7000 training trials with stimuli of equal duration, and only ∼25% accuracy after ∼9000 trials with the first stimulus eight times as long as the second (in this study, chance level was 16% (see electronic supplementary material, S1 and S2). The trace model also accounts for complex discriminations that are often analysed in terms of symbolic rules or grammars [17,21,23]. For example, zebra finch performance after training to respond to ABA and BAB but not to AAB, ABB, BAA or BBA (figure 1*h*) is accurately predicted by the trace model (figure 4*j*). Remarkably, the model also predicts correctly responding to untrained test sequences. For example, BBAA was treated like BAA (little responding), while BABA was treated like ABA (strong responding). This result is poorly explained in terms of rule or grammar learning [23], but follows naturally from the fact that the trace of sequence *X*_1_*X*_2_*X*_3_*X*_4_, where each *X*_*i*_ is either A or B, is bound to be similar to the trace of *X*_2_*X*_3_*X*_4_, because the memory of *X*_1_ in the first sequence is either overwritten, if the same stimulus reappears later in the sequence, or it is erased by the passage of time.

## 4. Discussion

Our results suggest that studying the ability to represent and process sequential information is important to understand cognitive differences between humans and other species. The hypothesis that sequential memory is more developed in humans seems supported by available evidence, but more work is needed to evaluate it conclusively. In particular, sequence discrimination experiments should be conducted with more species, as we discuss next.

Our sample (eight bird and five mammal species, including two primates) is sufficiently diverse to suggest widespread difficulties in sequence discrimination learning, but it does have gaps. Notably, we did not find experiments with apes. Endress *et al.* [35] found some differences between chimpanzees and humans in a habituation–dishabituation task with sound sequences, but the significance of this finding is unclear because the task did not require subjects to make a discrimination (that is, there was no consequence to responding or not responding to any of the sequences; this observation applies also to other habituation studies [36]). Apes perform similarly to other mammals in delayed match-to-sample experiments [34], but studies with other experimental paradigms have suggested good working memory at least for short intervals [37]. The latter studies, however, did not require the apes to remember sequences of stimuli, but rather arrays of simultaneously presented stimuli. In summary, much remains to be ascertained about the sequence-processing abilities of apes. Songbirds are another group that may have sequence representation abilities more advanced than a trace memory, although possibly restricted to song or other auditory stimuli. In most songbirds, juveniles learn to sing with the help of a template memory that is updated by listening to the song of adult birds [13,38]. How the template operates to guide song learning varies across species [13,39], but at least in some cases it seems capable of encoding accurate temporal information [40,41].

While our results suggests that humans have improved memory for sequential information, they do not show directly how human memory overcomes the limitations of non-human memory. One obvious possibility is that human memory is aided by language [42,43]. For example, language may enable the formation of explicit verbal strategies such as ‘Respond only when the first colour is blue and the second is yellow’. Although this possibility has intuitive appeal, it is also the case that language itself seems to require encoding and representation of sequential information. In other words, if we could not form concepts such as ‘first’, ‘second’, ‘before’ or ‘after’, we would not have words for them. It is also possible that neither language nor a faithful sequential memory are wholly primitive, and that they bootstrap each other during development. Indeed, research shows that sequence processing and language abilities are deeply intertwined [44–47]. Further studies charting the development of sequence discrimination abilities in pre-verbal and young children would be illuminating.

The accurate fit of a trace memory model to sequence discrimination data raises several issues for future research. The trace model is purely ‘retrospective’, i.e. decisions are taken at the time of responding based on ‘looking at the past’ through the (imperfect) lens of working memory. ‘Prospective’ accounts of animal working memory have also been proposed, in which decisions are taken (when logically possible) at the time stimuli are experienced, and are then remembered until it is time to respond [48,49]. Retrospective models have been sometimes rejected based on verbal arguments [48,49], but it may be worthwhile to re-evaluate the issue using computational models. Similar remarks can be offered with respect to other aspects of sequence information processing. For example, it has been suggested [50,51] that animals may form more sophisticated stimulus representations by grouping together sets of stimuli (‘chunking’; see [24]), or by representing stimuli in terms of abstract rules or grammars [17,23,52]. The fact that a trace model accounts well for many sequence discrimination tasks, including some that were originally devised as grammatical decision tasks [23,27], raises the possibility that a trace memory may also account for features of chunking, grammar learning, and possibly other paradigms. At the very least, a trace model can serve, in its simplicity, as a useful baseline to assess whether data warrant the assumption of more sophisticated forms of processing.

Our findings stand out among attempts to identify cognitive differences between humans and other animals: if non-human animals lack the capacity to faithfully represent sequential information, the difference between humans and animals may be more fundamental than it is often suggested. Current research, in fact, is focused on specific aspects of cognition such as planning [53,54], social learning [55–57], rule learning [17,58,59] or grammar learning [21,27,36,51,52]. Humans, however, excel in *all* these activities. Moreover, these activities all presuppose accurate encoding and representation of sequential information (table 1). Thus, a taxonomic gap in sequence encoding and representation would contribute significantly to explaining the cognitive divide between humans and other animals. If humans depended on simple memory traces for sequence processing, there would likely be no language, music, complex culture or mathematics on this planet.

## Supplementary Material

Supplementary information on data sources and methods

## Data accessibility

Data and code for model fitting are publicly available at https://doi.org/10.6084/m9.figshare.3179239.v1.
