# Supplementary material for: Memory for stimulus sequences: a divide between humans and other animals?
Source: R Soc Open Sci. 2017 Jun 21;4(6):161011. doi: 10.1098/rsos.161011 (PMC5493902; doi:10.1098/rsos.161011)
Supplement: Supplementary information on data sources and methods [file rsos161011supp1.pdf]

Supplementary Information for:
Ghirlanda S, Lind J, Enquist M, Memory for stimulus sequences: A divide between humans and other animals?

Stefano Ghirlanda   Johan Lind   Magnus Enquist

1 Summary of reviewed studies

We summarize below the information used for data analysis and figures in the main text. Each study is presented with a unique code (first author's last name + publication year + lowercase letter in case of ambiguity), bibliographic information, a summary, and a table that details the main aspects of experimental procedures and results. Each table has one line for each experiment reported in the study. The legend for the tables is as follows:

Species: The species on which the experiment was conducted.

Experiment: The experiment to which the data refers, as labeled in the original study.

Length: The number of stimuli in each stimulus sequence. Empty for non-quantifiable stimuli such as bird songs.

# Sequences: The number of sequences entering the discrimination task.

Trials: Average number of trials required to reach the performance level reported in the **Correct** column. Pre-training such as initial shaping of responses and single-stimulus training are not included as part of training. In some cases, the **Summary** for each study details further training information.

Correct: Average percentage of correct responses after the number of training trials given in the **Trials** columns.

Fit: Correlation coefficient between observed performance and predictions from the best-fitting trace memory model (see main text and Supplementary Information section 2 on fitting methods).

Stimuli: What kind of stimuli were used in the experiment. For brevity, the following codes are used:

- **R:** Red light
- **G:** Green light
- **B:** Blue light
- **W:** White light
- **Y:** Yellow light

P: Purple light

Light: Diffuse illumination of the experimental apparatus (e.g., overhead light)

Dark: A darkened experimental apparatus

Line: A line of a particular orientation

Pattern: A geometric visual pattern

Click: A click train

Tone: A pure tone

Note: A note from a musical instrument

Tune: A melody (used when several tone sequences of different length where employed)

Syllable: A human speech syllable

Song syllable: A unit of bird song

Song: A bird song. Song species is given if different from subject species

Sound: An auditory stimulus different from the ones listed above

S: Average stimulus duration within a sequence

ISI: Average inter-stimulus interval within a sequence

RI: Average retention interval, e.g., interval between the end of a sequence and the time at which the subject could respond

ITI: Average inter-trial interval

All durations are in seconds. When different durations where used, e.g., different ISIs within the same experiment, the average is reported. Complete information about each discrimination is in the data package https://figshare.com/s/70b4831ea543ea3d155b.

1. Aggleton2011

¿ sink()

Species	Experiment	Length	# Sequences	Trials	Correct	Fit	Stimuli	S	ISI	RI	ITI
Rat	1	2	6	720	0.54		Light, Tone, Click	10	0	0	60

Summary: Trained 3 *AB* vs. *BA* discriminations

Reference: J. P. Aggleton, E. Amin, T. E. Jenkins, J. M. Pearce, and J. Robinson. Lesions in the anterior thalamic nuclei of rats do not disrupt acquisition of stimulus sequence learning. *Quarterly Journal of Experimental Psychology*, 64(1):65–73, 2011

2. Bloomfield2008

Species	Experiment	Length	# Sequences	Trials	Correct	Fit	Stimuli	S	ISI	RI	ITI
Black-capped chickadee	BC-HR-BC	1	20	2500	0.8		Songs	2		0	
Black-capped chickadee	BC-HR-MO	1	20	4500	0.8		Songs	2		0	
Black-capped chickadee	BC-HR-PS	1	20	5300	0.8		Songs	2		0	
Black-capped chickadee	BC-WC-BC	1	20	3000	0.8		Songs	2		0	
Black-capped chickadee	BC-WC-MO	1	20	8500	0.8		Songs	2		0	
Black-capped chickadee	BC-WC-PS	1	20	8350	0.8		Songs	2		0	
Mountain chickadee	MC-HR-BC	1	20	2500	0.8		Songs	2		0	
Mountain chickadee	MC-HR-MO	1	20	3750	0.8		Songs	2		0	
Mountain chickadee	MC-HR-PS	1	20	6650	0.8		Songs	2		0	
Mountain chickadee	MC-WC-BC	1	20	2000	0.8		Songs	2		0	
Mountain chickadee	MC-WC-MO	1	20	2950	0.8		Songs	2		0	
Mountain chickadee	MC-WC-PS	1	20	6750	0.8		Songs	2		0	

Summary: Trained wild-caught (WC) and hand-raised (HR) black-capped chickadees (BC) and mountain chickadees (MC) to discriminate between two sets of 10 songs each. For some birds, rewarded songs where all BC, for others all MC, and for others still the rewarded and unrewarded songs were a mix of BC and MC songs (pseudo-category, PS).

Reference: L. Bloomfield, Laurie, T. M. Farrell, and C. B. Sturdy. Categorization and discrimination of "chick-a-dee" calls by wild-caught and hand-reared chickadees. *Behavioural Processes*, 77:166–176, 2008

3. Braaten1990

Species	Experiment	Length	# Sequences	Trials	Correct	Fit	Stimuli	S	ISI	RI	ITI
Starling	1/A	4	2	10700	0.75		Tones	<1	0	0	5
Starling	1/B	4	2	1940	0.75		Tones	<1	0	0	5
Starling	1/C	4	2	2510	0.75		Tones	<1	0	0	5
Starling	1/D	4	2	8020	0.75		Tones	<1	0	0	5
Starling	1/E	4	2	4880	0.75		Tones	<1	0	0	5
Starling	1/F	4	2	6010	0.75		Tones	<1	0	0	5

Summary: Exp. 1 trained starlings in six successive discriminations between one rising and one non-rising tone pattern. In tests with all patterns intermixed, performance decreased as a function of number discriminations learned thus far. Exp. 2–4 are about transfer of learning and are not analyzed here.

Reference: R. F. Braaten, S. H. Hulse, and S. C. Page. Discrimination and classification of rising and nonrising pitch patterns by the european starling (*Sturnus vulgaris*). *Animal Learning & Behavior*, 18(4):352–364, 1990

4. Braaten2007

Species	Experiment	Length	# Sequences	Trials	Correct	Fit	Stimuli	S	ISI	RI	ITI
Zebra finch	1	1	2	850	0.75		Songs	3		0	1
Zebra finch	2/CON	1	2	1270	0.75		Songs	3		0	1
Zebra finch	2/HET	1	2	1140	0.75		Songs	3		0	1

Summary: In Exp. 1, birds were trained to discriminate between two songs. The rewarded song was familiar, the other novel. Exp. 2 followed same procedure, but both zebra finch (CON) and starling (HET) songs were used.

Reference: R. F. Braaten, M. Y. Petzoldt, and A. K. Cybenko. Recognition memory for conspecific and heterospecific song in jhuvenile zebra finchs, *Taeniopygia guttata*. *Animal Behaviour*, 73:403–413, 2007

5. Braaten2008

Species	Experiment	Length	# Sequences	Trials	Correct	Fit	Stimuli	S	ISI	RI	ITI
Zebra finch	1/CON	1	2	740	0.75		Songs	3		0	1
Zebra finch	1/HET	1	2	630	0.75		Songs	3		0	1

Summary: Procedure similar to Braaten2007, but each zebra finch was exposed to both zebra finch and starling songs.

Reference: R. F. Braaten, S. S. Miner, and A. K. Cybenko. Song recognition memory in juvenile zebra finches: Effects of varying the number of presentations of heterospecific and conspecific song. *Behavioural Processes*, 77:177–183, 2008

6. Braaten2010

Species	Experiment	Length	# Sequences	Trials	Correct	Fit	Stimuli	S	ISI	RI	ITI
Zebra finch	1/IN	1	2	1520	0.75		Songs	3		0	1
Zebra finch	1/OUT	1	2	1160	0.75		Songs	3		0	1

Summary: Procedure similar to Braaten2007, but birds were exposed to songs either before (OUT) or during (IN) the sensitive period for song learning.

Reference: R. F. Braaten. Song recognition in zebra finches: Are there sensitive periods for song memorization? *Learning and Motivation*, 41:2012–212, 2010

7. Brown1971

Species	Experiment	Length	# Sequences	Trials	Correct	Fit	Stimuli	S	ISI	RI	ITI
Dog	1	2	4	490	0.83		Tones	3	0	0	60

Summary: Trained to discriminate *AB* and *BA* from *AA* and *BB*. Longer ISIs were trained after shorter ISIs.

Reference: B. L. Brown and S. Sołtysik. Four-pair same-different differentiation and transient memory in dogs. *Acta Neurobiologiae Experimentalis*, 31:87–100, 1971

8. Brown1971b

Species	Experiment	Length	# Sequences	Trials	Correct	Fit	Stimuli	S	ISI	RI	ITI
Dog	1	2	3	570	0.74		Tones	4	1	0	60

Summary: Trained to respond to *AB* and ignore *AA* and *BB*. Three dogs trained on multiple sequences failed to learn; these results are not included.

Reference:

9. Chen2015

Species	Experiment	Length	# Sequences	Trials	Correct	Fit	Stimuli	S	ISI	RI	ITI
Zebra finch	1a1	3	6	4450	NA	0.81	Song syllables	<1	0	0	
Zebra finch	1a2	3	6	1880	NA	0.99	Song syllables	<1	0	0	
Zebra finch	1a3	3	6	1370	NA	0.87	Song syllables	<1	0	0	
Zebra finch	1a4	3	6	1060	NA	0.97	Song syllables	<1	0	0	
Zebra finch	1a5	3	12	5640	NA	0.74	Song syllables	<1	0	0	
Zebra finch	1b	3	12	15030	NA	0.90	Song syllables	<1	0	0	
Human	2a	3	6	50	0.9		Song syllables	<1	0	0	
Human	2b	3	10	50	0.9		Song syllables	<1	0	0	

Summary: Trained zebra finches and humans on various sequence discrimination tasks involving sequences of three stimuli. The positive sequences followed an XYX pattern, while the negative ones a XXY pattern.

Reference: J. Chen, D. van Rossum, and C. ten Cate. Artificial grammar learning in zebra finches and human adults: XYX versus XXY. *Animal Cognition*, 18:151–164, 2015

10. DAmato1982

Species	Experiment	Length	# Sequences	Trials	Correct	Fit	Stimuli	S	ISI	RI	ITI
Capuchin	1	1	2	1940	0.83		Tunes			0	30
Rat	2	1	2	400	0.61		Tunes			0	30

Summary: Trained capuchin monkeys and rats to discriminate one 6-note tune from a 32 note ascending-descending gliss.

Reference: M. R. D'Amato and D. P. Salmon. Tune discrimination in monkeys (*Cebus apella*) and in rats. *Animal Learning & Behavior*, 10(2):126–134, 1982

11. DAmato1984

Species	Experiment	Length	# Sequences	Trials	Correct	Fit	Stimuli	S	ISI	RI	ITI
Capuchin	1/Monkey	1	2	820	0.9		Tunes	2		0	30
Rat	1/Rat	1	2	500	0.9		Tunes	2		0	30
Capuchin	2/Monkey	1	2	2520	0.9		Tunes	1		0	30
Rat	2/Rat	1	2	5350	0.9		Tunes	1		0	30

Summary: Exp. 1 trained rats and capuchin monkeys to discriminate between two tunes with different average frequency. Exp. 2 trained a similar discrimination, but with the tunes close in overall frequency. Data from two rats that failed to learn in Exp. 2 are not included. Data from the "random notes" conditions are not analyzed here. This condition involved many different tunes of unreported structure, composed at random from a given set of tones.

Reference: M. R. D'Amato and Salmon D. P. Processingof complext auditory stimuli (tunes) by rats and monkeys (*Cebus apella*). *Animal Learning & Behavior*, 12(2):184–194, 1984

12. Davis1986

Species	Experiment	Length	# Sequences	Trials	Correct	Fit	Stimuli	S	ISI	RI	ITI
Rat	1	2	3	3120	0.68		White noise	<1	1	0	

Summary: Trained to discriminate *AAA* from *AA* and *AAAA*.

Reference: H. Davis and M. Albert. Numerical discrimination by rats using sequential auditory stimuli. *Animal Learning & Behavior*, 14(1):57–59, 1986

13. DeLaMora2014

Species	Experiment	Length	# Sequences	Trials	Correct	Fit	Stimuli	S	ISI	RI	ITI
Rat	1	3	20	100	0.48		Notes, Syllables	<1	0	0	
Rat	2	3	32	100	0.53		Notes, Syllables	<1	0	0	

Summary: Exp. 1 trained a discrimination between 10 *AAB* piano note sequences vs. 10 *ABC* sequences. Exp. 2 trained structurally identical discriminations using 32 nonsense 3-syllable words.

Reference: D. De la Mora and J. M. Toro. Discrimination of acoustic patterns in rats using the water t-maze. *Psicológica*, 35(195-208), 2014

14. Dewson1969

Species	Experiment	Length	# Sequences	Trials	Correct	Fit	Stimuli	S	ISI	RI	ITI
Macaque	1	2	4	12720	0.9		Tones	<1	0	0	

Summary: Trained monkeys to discriminate between 4 2-tone sequences by performing a unique left/right lever pull sequence as a response. Trials do not include pre-training of about the same length on simpler tasks. Data on S and ISI variation presented in summary form unsuitable for model fitting, but qualitatively compatible with the memory trace model presented in the main text.

Reference: III Dewson, J. H. and A. Cowey. Discrimination of auditory sequences by monkey. *Nature*, 222:695–697, 1969

15. Dougherty1996

Species	Experiment	Length	# Sequences	Trials	Correct	Fit	Stimuli	S	ISI	RI	ITI
Pigeon	1	2	2	3250	0.72		RGB	5	8	0	15

Summary: Trained to discriminate *AB* from *B*.

Reference: D. H. Dougherty and J. T. Wixted. Detecting a nonevent: delayed presence-versus-absence discrimination in pigeons. *Journal of the Experimental Analysis of Behavior*, 65:81–92, 1996

16. Gentner2006

Species	Experiment	Length	# Sequences	Trials	Correct	Fit	Stimuli	S	ISI	RI	ITI
Starling	1	4	16	25000	0.58		Starling song syllables	<1	0	0	

Summary: Trained to discriminate 8 *ABBA* sequences from 8 *ABAB* sequences.

Reference: Timothy Q Gentner, Kimberly M Fenn, Daniel Margoliash, and Howard C Nusbaum. Recursive syntactic pattern learning by songbirds. *Nature*, 440(7088):1204–1207, 2006

17. Ghirlanda2016

Species	Experiment	Length	# Sequences	Trials	Correct	Fit	Stimuli	S	ISI	RI	ITI
Human	1	2	4	100	1		BY	1		0	3

Summary: Replication of Weisman1980 under more challenging conditions with shorter stimulus duration and inter-trial interval.

Reference:

18. Hulse1984

Species	Experiment	Length	# Sequences	Trials	Correct	Fit	Stimuli	S	ISI	RI	ITI
Starling	1	1	8	5800	0.9		Tones	4		0	5

Summary: Starlings were trained to discriminate 4 rising tone sequences from 4 falling sequences. Performance is estimated from response latency data.

Reference: Stewart H. Hulse, John Humpal, and Jeffrey Cynx. Discrimination and generalization of rhytmic and arrythmic sound patterns by european starlings (*Sturnus vulgaris*). *Music Perception*, 1(4):442–464, 1984

19. Izumi2002

Species	Experiment	Length	# Sequences	Trials	Correct	Fit	Stimuli	S	ISI	RI	ITI
Macaque	1	2	12	1920	0.89		Tones	2	1	0	3

Summary: Trained to discriminate rising vs. non-rising series of tones. The monkeys had extensive previous experience from similar tasks. Only data without simultaneous interfering tones is analyzed here.

Reference: Akihiro Izumi. Auditory stream segregation in japanese monkeys. *Cognition*, 82(3):B113–B122, 2002

20. Izumi2003

Species	Experiment	Length	# Sequences	Trials	Correct	Fit	Stimuli	S	ISI	RI	ITI
Human	1/Human	4	4	120	0.93		Tones	<1	1	0	3
Macaque	1/Macaque	4	4	1710	0.7	0.69	Tones	<1	1	0	3
Macaque	2	5	24	3820	0.72		Tones		0	0	3

Summary: Trained to discriminate between rising and falling sequences of 2–3 tones. S was 100–300 ms for macaques, 10–50 ms for humans. Exp. 2 used the same macaques. The monkeys had extensive previous experience on similar tasks. Data from Exp. 2 were not fit because of little variation in performance across sequences (5% variation for one monkey, 15% for the other).

Reference: Akihiro Izumi. Effect of temporal separation on tone-sequence discrimination in monkeys. *Hearing research*, 175(1):75–81, 2003

21. Kudoh2004

Species	Experiment	Length	# Sequences	Trials	Correct	Fit	Stimuli	S	ISI	RI	ITI
Rat	1	10	2	7000	0.65		Tone,Sound	<1	1	0	60

Summary: Trained to dscriminate *AB* from *BA*. Each sequence was repeated 5 times, separated by about 1.5 s.

Reference: Masaharu Kudoh, Kenjiro Seki, and Katsuei Shibuki. Sound sequence discrimination learning is dependent on cholinergic inputs to the rat auditory cortex. *Neuroscience Research*, (113-123), 2004

22. Kudoh2006

Species	Experiment	Length	# Sequences	Trials	Correct	Fit	Stimuli	S	ISI	RI	ITI
Rat	1	2	2	8000	0.75		Tone, Sound		0	0	

Summary: Same training as Kudoh2004.

Reference: Masaharu Kudoh and Katsuei Shibuki. Sound sequence discrimination learning motivated by reward requires dopaminergic D2 receptor activation in the rat auditory cortex. *Learning & Memory*, 13:690–698, 2006

23. MacDonald1993

Species	Experiment	Length	# Sequences	Trials	Correct	Fit	Stimuli	S	ISI	RI	ITI
Pigeon	1	2	6	6620	0.47	0.91	RGY	5	0	0	
Pigeon	2	2	6	7490	0.38	0.86	RGY	4	0	0	
Pigeon	3	2	6	9220	0.26	0.95	RGY	4	0	0	
Pigeon	4	2	6	9500	0.33	0.85	RGY	4	0	0	

Summary: Trained to reproduce sequences of 2 coloured lights by pecking the lights themselves in correct order, after the sequence had been presented. The different experiments manipulate the relative duration of the two lights, as well as ISI and RI intervals. Pigeons in Exp.n had been part of Exp.$(n-1)$. The author claims results falsify the memory trace model, but in fact the model fits well, see Figure 3 in the main text.

Reference: S. E. MacDonald. Delayed matching-to-successive-samples in pigeons: Short term memory for item and order information. *Animal Learning & Behavior*, 21:59–67, 1993

24. Murphy2004

Species	Experiment	Length	# Sequences	Trials	Correct	Fit	Stimuli	S	ISI	RI	ITI
Rat	1/a	2	8	1680	0.51		Light/Dark, Tones	10	1	0	68
Rat	1/b	2	8	2080	0.52		Light/Dark, Tones	6	1	0	68

Summary: Trained dicriminations between 4 sequences and their reversals. Exp. 2 required additional training (amount not reported).

Reference: R. A. Murphy, E. MOndragón, V. A. Murphy, and N. Fouquet. Serial order of conditional stimuli as a discriminative cue for Pavlovian conditioning. *Behavioural Processes*, 67:303–311, 2004

25. Murphy2008

Species	Experiment	Length	# Sequences	Trials	Correct	Fit	Stimuli	S	ISI	RI	ITI
Rat	1	3	6	1440	0.52		Light/Dark	10	0	0	60
Rat	2	3	6	1350	0.57		Tones	10	0	0	60

Summary: Trained discrimination between 2 positive and 4 negative sequences. Two rats failed to learn in Exp. 2, excluded from our analysis.

Reference: R. A. Murphy, E. Mondragón, and V. A. Murphy. Rule learning by rats. *Science*, 319:1849–1851, 2008

26. Nelson1978

Species	Experiment	Length	# Sequences	Trials	Correct	Fit	Stimuli	S	ISI	RI	ITI
Pigeon	1	2	4	19600	0.73	0.96	RG	5	6	0	25
Pigeon	2	2	4	22600	0.68	0.97	RG	5	11	0	27
Pigeon	3	2	4	6000	0.58	0.96	RG	5	11	0	25

Summary: Trained to discriminate *AA* and *BB* from *AB* and *BA*. Authors labeled RI what we label ISI. Some birds received additional training. Exp. 2 used 2 birds from Exp. 1.

Reference: Keith R. Nelson and Edward A. Wasserman. Temporal factors influencing the pigeon's successive matching-to-sample performance: Sample duration, intertrial interval, and retention interval. *Journal of the Experimental Analysis of Behavior*, 30:153–162, 1978

27. Roitblat1987

Species	Experiment	Length	# Sequences	Trials	Correct	Fit	Stimuli	S	ISI	RI	ITI
Pigeon	1	3	27	17280	0.69	0.71	RGB	1	0	5	18

Summary: Trained to discriminate *ABC* from the other possible combinations of *A*, *B*, and *C*. Model fit based on data from block 10, except for subject B939, for which block 20 as used as discrimination at block 10 was at chance level.

Reference: H. L. Roitblat, R. A. Scopatz, and T. G. Bever. The hierarchical representation of three-item sequences. *Animal Leanring & Behavior*, 15(2):179–192, 1987

28. Roitblat1991

Species	Experiment	Length	# Sequences	Trials	Correct	Fit	Stimuli	S	ISI	RI	ITI
Pigeon	1/LSS	3	27	25920	0.59		RGB	1	1	0	15
Pigeon	1/MMM	3	27	25920	0.66		RGB	1	1	0	15
Pigeon	1/SSL	3	27	25920	0.65		RGB	1	1	0	15

Summary: Trained *ABC* vs. other combinations of *A*, *B*, and *C*. Training trials estimated from Fig. 1.

Reference: H. L. Roitblat, T. G. Bever, D. A. Hedweg, and H. E. Harley. On-line choice and the representation of serially structured stimuli. *Journal of Experimental Pscyhology: Animal Behavior Processes*, 17(1):55–67, 1991

29. Sambeth2006

Species	Experiment	Length	# Sequences	Trials	Correct	Fit	Stimuli	S	ISI	RI	ITI
Human	1-Human	2	2	10	1		Light, Tone	1	5	0	12
Rat	1-Rat	2	2	480	0.76		Light, Tone	5	5	0	120

Summary: Trained humans and rats on a *AB* vs. *B* discrimination.

Reference: A. Sambeth and J. H. R. Maes. A comparison of event-related potentials of humans and rats elicited by a serial feature-positive discrimination task. *Learning and Motivation*, 37:269–288, 2006

30. Seki2013

Species	Experiment	Length	# Sequences	Trials	Correct	Fit	Stimuli	S	ISI	RI	ITI
Bengalese finch	1	3	10	6180	0.78		Finch calls, ‘na’ syllable	<1	0	0	4
Human	2/BV	3	10	50	0.98		Finch calls, ‘na’ syllable	<1	0	0	0
Human	2/HV	3	10	60	0.98		Finch calls, ‘na’ syllable		0	0	0

Summary: Trained Bengalese finches to discriminate 5 *AAB* sequences from 5 *ABB* sequences. Also trained human subjects on the same discriminations, with either finch syllables of “na” speech syllable spoken by a male or female.

Reference: Yoshimasa Seki, Kenta Suzuki, Ayumi M Osawa, and Kazuo Okanoya. Songbirds and humans apply different strategies in a sound sequence discrimination task. *Frontiers in psychology*, 4, 2013

31. Shimp1974

Species	Experiment	Length	# Sequences	Trials	Correct	Fit	Stimuli	S	ISI	RI	ITI
Pigeon	1	2	4	10000	0.84	0.91	RBW	<1	1	2	1
Pigeon	2	3	7	22500	0.79	0.92	RBW	<1	0	2	1

Summary: Trained to peck the key on which the 1st, 2nd, or 3rd stimulus of a 3-stimulus sequence had appeared. $\sim$15000 pre-training trials not included in learning trials. Exp. 2 used the same pigeons as Exp. 1.

Reference: C. P. Shimp and M. Moffitt. Short-term memory in the pigeon: Stimulus-response associations. *Journal of the Experimental Analysis of Behavior*, 22:507–512, 1974

32. Shyan1987

Species	Experiment	Length	# Sequences	Trials	Correct	Fit	Stimuli	S	ISI	RI	ITI
Macaque	1	2	2	1480	0.8		Sounds	12	0	0	25

Summary: Trained one macaque on a delayed same-different discrimination with 38 sounds as stimuli. The macaque had previously failed to learn the task in ∼17000 trials with a different technique.

Reference: M. R. Shyan, A. A. Wright, R. G. Cook, and M. Jitsumory. Acquisition of the auditory *same/different* task in a rhesus monkey. *Bulleting of the Pscyhonomic Society*, 25(1):1–4, 1987

33. Spierings2016

Species	Experiment	Length	# Sequences	Trials	Correct	Fit	Stimuli	S	ISI	RI
Budgerigar	B	3	6	7820	0.9	0.73	Zebra finch song syllables, artificial sounds	<1	0	0
Zebra finch	ZF1	3	6	13850	0.86	0.61	Zebra finch song syllables, artificial sounds	<1	0	0
Zebra finch	ZF2	3	6	13240	0.9	0.71	Zebra finch song syllables, artificial sounds	<1	0	0

Summary: In Exp. ZF1, zebra finches were trained to respond to 5 *ABA* sequences, and not to respond to 5 *AAB* sequences composed of the same sounds. The birds were then tested with other sequences in *ABA* and *AAB* pattern. Exp. ZF2 was a replication with artificial sounds rather than zebra finch song syllables. Exp. B was a replication of Exp. ZF1 with budgerigars as subjects.

Reference:

34. Strominger1980

Species	Experiment	Length	# Sequences	Trials	Correct	Fit	Stimuli	S	ISI	RI	ITI
Macaque	1	3	2	300	0.9		Tones		0	0	

Summary: Trained monkeys on a single *ABA* vs. *BAB* discrimination.

Reference: N. L. Strominger, R. E. Oesterreich, and W. D. Neff. Sequential auditory and visual discrimination after temporal lobe ablation in monkeys. *Physiology & Behavior*, 24:1149–1156, 1980

35. Thompson1976

Species	Experiment	Length	# Sequences	Trials	Correct	Fit	Stimuli	S	ISI	RI	ITI
Dolphin	B	2	2	810	0.89		Sounds	1	0	0	
Dolphin	C	2	4	770	0.9		Sounds	1	0	0	
Dolphin	Di	2	4	1060	0.94		Sounds	1	0	0	
Dolphin	Dii	2	4	1160	0.89		Sounds	1	0	0	
Dolphin	E	2	4	1930	0.94		Sounds	1	0	0	
Dolphin	F	2	4	2330	0.96		Sounds	1	0	0	

Summary: First trained *BC* vs. *BD*, then added *AC* vs. *AD*. Some trials and experimental phases where "cued" by the addition of a previously rewarded stimulus; these are not analyzed here.

Reference: R. K. R. Thompson. *Performance of the bottlenose dolphin (Tursiops truncatus) on delayed auditory sequences and delayed auditory successive discriminations*. PhD thesis, University of Hawaii, 1976

36. Wasserman1980

Species	Experiment	Length	# Sequences	Trials	Correct	Fit	Stimuli	S	ISI	RI	ITI
Pigeon	1	3	16	6000	0.78	0.90	RGBYV, Lines	2	0	0	20
Pigeon	2	3	4	8000	0.69	0.98	RGBYV, Lines	2	1	0	20

Summary: Trained to discriminate between 16 possible sequences (4 positive). Exp. 1 trials in the table concern the fastest learners, some pigeons required 11600 trials. Exp. 2 used the same pigeons.

Reference: E. A. Wasserman, K. R. Nelson, and M. B. Larew. Memory for sequences of stimuli and responses. *Journal of the Experimental Analysis of Behavior*, 34:49–59, 1980

37. Watanabe1981

Species	Experiment	Length	# Sequences	Trials	Correct	Fit	Stimuli	S	ISI	RI	ITI
Macaque	colorI	2	4	80000	0.95		RG, Pattern	1		2	5
Macaque	colorII	2	4	20000	0.95		RG, Pattern	1		2	5
Macaque	pattern	2	4	20000	0.95		RG, Pattern	1		2	5

Summary: Trained to discriminate *AXCX* from *BXCX*, where *A* and *B* were red or green lights, *X* was blank and *C* was the presentation of two patterns (one had to be touched to get the reward). Trials given are an underestimate; the author reports 1000 daily trials for 4 months.

Reference: M. Watanabe. Prefrontal unit actiity during delayed conditional discriminations in the monkey. *Brain Research*, 225:51–65, 1981

38. Watanabe1986

Species	Experiment	Length	# Sequences	Trials	Correct	Fit	Stimuli	S	ISI	RI	ITI
Macaque	CC	2	4	40000	0.95		RG, patterns	1		2	5
Macaque	CP	2	4	120000	0.95		RG, patterns	1		2	5
Macaque	PP	2	4	40000	0.95		RG, patterns	1		2	5

Summary: Exp. CP as Exp. colorI in Watanabe1981. Two subjects also completed Exp. CC and PP, which were *AB* vs. *BB* discriminations.

Reference: M. Watanabe. Prefrontal unit activity during delayed conditional Go/No-Go discrimination in the monkey. I. Relation to the stimulus. *Brain Research*, 382:1–14, 1986

39. Weary1991

Species	Experiment	Length	# Sequences	Trials	Correct	Fit	Stimuli	S	ISI	RI	ITI
Black-capped chickadee	1	2	4	16150	0.8		Song syllables	<1	0	0	

Summary: Trained to discriminate 2 positive from 2 negative 2-syllable sequences. Exp. 2 included no new discriminations and is not analyzed here.

Reference: D. M. Weary and R. G. Weisman. Operant discrimination of frequency and frequency ratio in the black-capped chickadee (*Parus atricapillus*). *Journal of Comparative Psychology*, 105(3):253–259, 1991

40. Weisman1980

Species	Experiment	Length	# Sequences	Trials	Correct	Fit	Stimuli	S	ISI	RI	ITI
Pigeon	1K	2	4	3510	0.92	0.94	RGB, Lines	5		0	35
Pigeon	1O	2	4	3510	0.88	0.80	RGB, Lines	5		0	35
Pigeon	2G	2	4	5400	0.71	0.77	RGB, Lines		0	0	35
Pigeon	3	2	8	4800	0.76	0.91	RGB, Lines	2		0	15

Summary: Exp. 1 trained pigeons in *AB* vs. *AA*, *BA*, *BB*. In Exp. 2, *C* was added in a generalization test. In Exp. 3, correct sequence included a 3 extsuperscriptrd stimulus, a horizontal or vertical line. The fit to Exp. 3 data is for day 16 in the case of subject sB12 nd B42, which equates the amount of training to that of other birds (15 days), as much as possible given available data.

Reference: RG Weisman, EA Wasserman, PW Dodd, and Mark B Larew. Representation and retention of two-event sequences in pigeons. *Journal of Experimental Psychology: Animal Behavior Processes*, 6(4):312, 1980

41. Weisman1981

Species	Experiment	Length	# Sequences	Trials	Correct	Fit	Stimuli	S	ISI	RI	ITI
Pigeon	1	2	5	15550	0.89		RY	5		2	8
Pigeon	2	2	5	24190	0.83		RY	3		0	8

Summary: Trained *AB* vs. *BA*, *BB*, *AA*, and no stimulus. Same subjects in both experiments.

Reference: RG Weisman and MP DiFranco. Testing models of delayed sequence discrimination in pigeons: Delay intervals and stimulus durations. *Journal of Experimental Psychology: Animal Behavior Processes*, 7(4):413, 1981

42. Weisman1985

Species	Experiment	Length	# Sequences	Trials	Correct	Fit	Stimuli	S	ISI	RI	ITI
Pigeon	1	3	8	5820	0.82	0.99	RY	3	0	0	8
Pigeon	2/Base	3	8	7820	0.8	0.96	RY	3	0	0	8
Pigeon	2/ISI1	3	8	7820	0.67	0.97	RY	3	3	0	8
Pigeon	2/ISI2	3	8	7820	0.71	0.96	RY	3	3	0	8
Pigeon	2/RI	3	8	7820	0.75	0.96	RY	3	0	5	8

Summary: Trained *BAB* vs. 7 other 3-stimulus sequences. Trials given for Exp. 1: trials are for the fastest learners, some birds required 14280 trials. Figure 1 in the main text shows the first 3500 trials. Exp. 2: trials are approximate because of missing details. Same pigeons in both experiments.

Reference: R. G. Weisman, C. Duder, and R. von Konigslow. Representation and retention of three-event sequences in pigeons. *Learning and Motivation*, 16:239–258, 1985

43. Weisman1987

Species	Experiment	Length	# Sequences	Trials	Correct	Fit	Stimuli	S	ISI	RI	ITI
Rat	1DCD	2	4	1200	0.67	0.99	Light, Dark, Tone, Click	8	2	0	20
Rat	1DD	2	4	1200	0.87	1.00	Light, Dark, Tone, Click	8	2	0	20
Rat	2DCD	2	4	2350	0.73	1.00	Light, Dark, Tone, Click	8	5	0	20
Rat	2DD	2	4	2350	0.88	0.94	Light, Dark, Tone, Click	8	11	0	20

Summary: Trained rats to discriminate *AC* and *AD* from *BC* and *AC* (Exp. 1) or *AC* and *BD* from *AD* and *BC* (Exp. 2). Exp. 2 used the same rats. Data after administration of schopolamine not analyzed here.

Reference: R. G. Weisman, R. Bruce, and R. J. Beninger. Simple and conditional discriminations in rats: The effects of delays and scopolamine. *Learning and Motivation*, 18:274–287, 1987

44. Wilson1985

Species	Experiment	Length	# Sequences	Trials	Correct	Fit	Stimuli	S	ISI	RI	ITI
Pigeon	1	2	4	2280	0.66		Food, Lights	3	12	0	25
Jackdaw	2/Jackdaw	2	4	1800	0.85		Food, Lights	3	14	0	20
Pigeon	2/Pigeon	2	4	1800	0.63		Food, Lights	3	14	0	20

Summary: Trained to peck one color if food had been previosuly delivered, another color if food had not been delivered. Food/No-food stimulus was followed by a variable delay of 2–38 s. Jackdaws performed better than pigeons at intervals up to 20 s, with a similar amount of training.

Reference: A. C. Wilson. The molecular basis of evolution. *Scientific American*, 253(4):148–157, 1985

45. vanHeijningen2013

Species	Experiment	Length	# Sequences	Trials	Correct	Fit	Stimuli	S	ISI	RI	ITI
Zebra finch	1	3	6		0.76	0.89	Song syllables	<1	0	0	

Summary: Exp. 1 trained to respond to *ABA* and *BAB* and ignore *AAB*, *ABB*, *BAA*, and *BBA*. Two birds did not meet 75% correct criterion. Exp. 2 added *ABBA* and *BAAB* as positive and *ABAB*, *AABB*, *BABA*, and *BBAA* as negative sequences. Only two birds reached criterion. The latter results are not analyzed here

Reference: Caroline A. A. van Heijningen, Jiani Chen, Irene van Laatum, Bonnie van der Hulst, and Carel ten Cate. Rule learning by zebra finches in an artificial grammar learning task: which rule? *Animal Cognition*, 16(2):165–175, 2013

2 Model fitting

The memory trace model described in the main text (equations 1 and 3) states that responding to a stimulus sequence x is a linear function of the sequence's Euclidean distance from positive and negative sequences, i.e., from sequences the animal has been trained to respond (positive) or to not respond (negative). Fitting this model to data means finding the values of the memory parameters r_{up}, r_{down}, and r_{blank}(see main text) that best account for the data. Below, we first describe how we evaluate model fit for a given set of parameter values, and then how we search for those parameter values that maximize the fit. We conclude with some further considerations about the fitting procedure.

2.1 Calculating model fit for a given set of parameters

In this section we assume that we have given values of the three parameters r_{up}, r_{down}, and r_{blank}, and we show how to compute the fit between the model and a given data set. A generic experiment involves $p \geq 1$ positive sequences $y_1, \ldots, y_p$ and $n \geq 1$ negative sequences $z_1, \ldots, z_n$. According to equation 3 in the main text, the model's prediction regarding responding to any sequence x is:

$$R(x) = g + h\left(\frac{1}{p}\sum_{i=1}^{p} d(x, y_i) - \frac{1}{n}\sum_{i=1}^{n} d(x, z_i)\right) \tag{1}$$

where we made explicit the averaging operation indicated as $\langle \cdot \rangle$ in the main text. The distances $d(x, y_i)$ and $d(x, z_i)$ should be evaluated at the time, $t^\star$, when the animal's response is recorded.

To calculate the distances in equation 1, we need to calculate how the sequences x, y_i ($i = 1, \ldots, p$), and z_i ($i = 1, \ldots, n$) are represented in memory. If the experiment involves m stimuli, $S_1, \ldots, S_m$, the memory representation of a sequence is an m-dimensional vector whose i-th component is the value of the memory trace of stimulus S_i, which we need to evaluate time $t^\star$, as mentioned above. We write the value of the memory trace of S_i at $t^\star$ as $m_{S_i}(t^\star)$. This value can be computed from equation 1 in the main text, given that the times when stimuli are presented are known as part of the experimental design. We rewrite the equation here for convenience:

$$m'_{S_i}(t) = \begin{cases} r_{\text{up}}\left[1 - m_S(t)\right] & \text{if } S_i \text{ is present} \\ -r_{\text{down}} m_S(t) & \text{if } S_i \text{ is absent} \end{cases} \tag{2}$$

(If no other stimulus is present when S is absent, $r_{\text{down}}$is replaced by r_{blank}, see main text.) Equation 2 is a first-order ordinary differential equation with constant coefficients and piecewise-constant input that can be solved by standard methods. For example, if S_i is present between t_{on} and t_{off}, the solution at time $t^\star$ is:

$$m_{S_i}(t^\star) = \begin{cases} 0 & t^\star < t_{\text{on}} \\ 1 - e^{-r_{\text{up}}(t^\star - t_{\text{on}})} & t_{\text{on}} \leq t^\star < t_{\text{off}} \\ \left(1 - e^{-r_{\text{up}}(t_{\text{off}} - t_{\text{on}})}\right) e^{-r_{\text{down}}(t^\star - t_{\text{off}})} & t^\star \geq t_{\text{off}} \end{cases} \tag{3}$$

Let us write $\mathbf{m}(x, t^\star)$ the vector of memory traces at time $t^\star$ after the presentation of sequence x:

$$\mathbf{m}(x, t^\star) = \left(m_{S_1}(x, t^\star), \ldots, m_{S_m}(x, t^\star)\right) \tag{4}$$

We can now calculate the distance between the representations of sequences x and y at $t^\star$ as:

$$d(x, y) = \sqrt{\sum_{i=1}^{s} \left[m_{S_i}(x, t^\star) - m_{S_i}(y, t^\star)\right]^2} \tag{5}$$

Repeating this calculation for all distances $d(x, y_i)$ and $d(x, z_i)$ that appear in equation 1, we can calculate the values:

$$D(x) = \frac{1}{p}\sum_{i=1}^{p} d(x, y_i) - \frac{1}{n}\sum_{i=1}^{n} d(x, z_i) \tag{6}$$

These values determine $R(x)$ as $R(x) = g + hD(x)$, but we can use them directly in model fitting without concerning ourselves with the values of h and g. Namely, let $x_i, \ldots, x_s$ be the sequences presented in the experiment, and let R_i be the observed response to x_i. Using the procedure detailed above, we calculate the $D(x_i)$ values for all such sequences, and then we quantify the fit, f, between the values $D(x_i)$ and R_i as

$$f(r_{\text{up}}, r_{\text{down}}, r_{\text{blank}}) = \text{cor}(D(x_i), R_i) \tag{7}$$

where $\text{cor}(\cdot,\cdot)$ denotes Pearson's correlation. Because linear transformations leave correlations unaffected, it does not matter whether we use $D(x_i)$ or $R(x_i)$. Note that, on the r.h.s. of equation 7, the dependence on model parameters r_{up}, r_{down}, and $r_{\text{blank}}$is implicit, but it exists as the distances that enter the $D(x_i)$ values are based on memory traces, which are affected by the value of r_{up}, r_{down}, and r_{blank}.

2.2 Maximizing model fit

In the previous section we showed how to calculate how well a given parameter triplet $\rho = (r_{\text{up}}, r_{\text{down}}, r_{\text{blank}})$ fits a set of experimental data. The last step of model fitting is to find the triplet that best fits the data. To do this, we maximize the correlation in equation 7 by systematic exploration of the parameter space. That is, we define lower and upper boundaries for r_{up}, r_{down}, and r_{blank}, and we evaluate model fit on all point of a three-dimensional lattice of equally spaced points lying within these boundaries. We then select the parameter triplet that yields the highest fit, which is the final result of model fitting. The range of values delimiting our search space was generally $0.1 - -10\,s^{-1}$ for all three parameters, with the constraint $r_{\text{blank}} \leq r_{\text{down}}$.

Once we have determined the best fitting values of r_{up}, r_{down}, and r_{blank}, we fit g and h in equation 1 using a linear model, to match numerically the observed response values. The values plotted in Figure 4 in the main text are the values of $R(x_i)$ with the best fitting g, h, r_{up}, r_{down}, r_{blank}. Note however, that fitting g and h does not affect model fit, as remarked above. Rather, it serves merely to transform the $D(x_i)$ values so that they lie within the same range as the observed R_i values, which is convenient for ease of comparison. A $D(x_i)$ value, in fact, has arbitrary units (representing distance in an abstract memory space), while responses R_i have units such as rate of responding or fraction of trials in which a response was observed.

2.3 Further considerations

To fit the model to data, we had to construct an empirical metric of the difficulty of discriminations. The best such metric would be to have learning curves for all discriminations involved in a study, but what is most often available is performance on each discrimination at the end of training. Fortunately, at any given time, learning speed and performance correlate, because the discrimination that proceeds more quickly has, by definition, the highest performance (see Figure 1 in the main text). Therefore, we can use performance at a given time to gauge the relative difficulty of discriminations. Most often, we use data on performance at the end of training. In a few cases, however, training continues for long enough that the performance on many discriminations is almost equally good. In these cases, we use, when available, data from earlier stages of training.

The general procedure just described had, in some cases, to be adapted to accommodate missing data or to address specific features of a study. For example, the model predicts short inter-trial intervals (ITIs) to be detrimental to responding because a short ITI may not allow the memory of a trial to decay fully before the next trial starts, which effectively decreases the distance between memory traces. Lacking precise information about the succession of trials, we estimated the memory trace at the beginning of each trial by calculating the trace that would be left, after the ITI, by each sequence appearing in the experiment, and then using the average of these traces as the initial memory trace for each trial. This is adequate if trial order is randomized or pseudo-randomized, as is typical in the reviewed experiments.

In Spierings2016, only responses to a subset of training sequences are reported. We assumed responses to all

training sequences to be the same as the reported ones.

Another case in which we modified the fitting procedure slightly concerns data from MacDonald1993 (see section 1 of this Supplementary Information). In her experiments, pigeons had to produce a sequence of two pecks. Namely, pigeons were shown a sequence of two colors, and then asked to reproduce the sequence by pecking at two of three key lights, simultaneously lit with the colors that had been just shown as well as a third color. In this case, we computed distances between memory traces at the time of both the first and second peck (assumed to occur 5 s later based on information about stimulus duration). We assumed that the decision to peck a color first would depend on the distance between the memory trace of the sequence just witnessed and the memory traces of the rewarded and unrewarded sequences having the color in first position. For example, suppose the sequence *AB* is showed and, successively, the pigeon has to choose which to peck among the colors *A*, *B*, and *C*. In such a situation the pigeon would choose to peck *A* first if it remembered (correctly) having seen *AB*, but also if it remembered (incorrectly) having seen *AC*. Conversely, the pigeon would choose not to peck *A* if it remembered to have seen any of the sequences without *A* in first position, namely *BA*, *BC*, *CA*, or *CB*. A similar argument holds for the probabilities to choose other stimuli. The choice of which color to peck second is determined in the same way, but using the memory traces at the time of the second choice. Overall, this reasoning still gives rise to equation 3 in the main text as a means to related a sample sequence to rewarded and non-rewarded sequences, but the sets of rewarded and non-rewarded sequences are different for each sample sequence.

Lastly, it would be valuable to obtain confidence intervals for our estimates of r_{up}, r_{down}, and r_{blank}, which are in practice estimates of animal memory spans under given experimental conditions. However, we have not pursued this line of investigation for several reasons. First, our main goal was not to quantify memory spans accurately, but to show that a memory model with an imperfect representation of order accounts well for animal data. Second, memory spans are observed to differ across experimental conditions (cf. J. Lind, S. Ghirlanda, and M. Enquist. Animal memory: A review of delayed match-to-sample data from 25 species. *Behavioral Processes*, 117:52–58, 2015). Thus, while estimates of r_{up}, r_{down}, and r_{blank}would be valid under specific experimental conditions, a useful quantification of memory span would require a systematic investigation of how these parameters vary across studies. It was not our aim to undertake such an investigation. Furthermore, distances between memory traces depend non-linearly on r_{up}, r_{down}, and r_{blank}, so that estimating their confidence interval is not trivial.

3 Human sequence discrimination experiment

To compare human and non-human memory for sequences, we replicated Experiment 1 in "RG Weisman, EA Wasserman, PW Dodd, and Mark B Larew. Representation and retention of two-event sequences in pigeons. *Journal of Experimental Psychology: Animal Behavior Processes*, 6(4):312, 1980." In this experiment, pigeons were trained to peck a white square if they had just seen an *AB* sequence of lights, and to refrain from pecking after *BA*, *AA*, and *BB* sequences. The results are shown in Fig. 1d in the main text. In our experiments, human subjects received these instructions on screen:

> The data collected in this experiment will not be linked to your name or other identifying information. If you would like to terminate the experiment at any time, you may do so without penalty.
>
> PLEASE READ THE FOLLOWING INSTRUCTIONS CAREFULLY.

THE EXPERIMENT LASTS LESS THAN 15 MINUTES, but if you don't pay attention your results may mislead us to incorrect conclusions! PLEASE TRY TO PAY ATTENTION THROUGHOUT THE EXPERIMENT!

During the experiment you will see short sequences of colored squares. The last square of each sequence will always be white. When you see the white square, you have the option of doing nothing or pressing the spacebar. Pressing the spacebar to certain sequences will cause a smiley face to appear. This means that your response was correct. However, pressing the spacebar to other sequences will not cause a smiley face to appear. For these sequences, you should choose to do nothing.

You have to learn which sequences will cause a smiley face to appear when you press the spacebar. Initially, of course, you will not know when to press, so you may respond incorrectly. This is fine, but try to press the spacebar only if you think that doing so will make the smiley face appear.

You must decide quickly, because the white square will be shown only briefly. If you do not respond fast enough, the experiment will move on. Responding before the white square appears is a mistake and will be ignored.

If you are clear on the above instructions, you can press 'Y' now to begin the experiment.

The explicit instruction to respond only to the white square mirrors the pigeons' preliminary training to peck only at a white square (the length of this training is not included in our analysis). For half of our participants, the correct sequence was yellow-blue, for the other half it was blue-yellow. By nonhuman standards (Fig. 2b in the main text), our replication is more difficult than the original experiment because it features shorter stimuli (1 s rather than 5 s) and inter-trial intervals (3 s rather than 35 s). The experiment was programmed using the ALEX software, freely available at www.github.com/drghirlanda/alex. The configuration files are available upon request. The experiment was approved by the CUNY IRB with code 412807.

Thirty-nine participants were recruited from the subject pool of the Brooklyn College Department of Psychology and participated for course credit. The latter was not contingent upon performance. Nine participants either never responded, or responded on every trial (presumably to finish the experiment earlier). We excluded these data from analysis. The results from the remaining subjects appear alongside the pigeon data in Fig. 1d in the main text.
